# Ionic Liquid/Deep Eutectic Solvent-Mediated Calcining Synthesis of Cobalt-Based Electrocatalysts for Water Splitting

**DOI:** 10.3390/molecules29184435

**Published:** 2024-09-18

**Authors:** Chenyun Zhang, Jianjiao Jin, Jiahao Wang, Fangfang Sun, Jiacheng Xu, Shun Wang, Lihua Xu, Jing Zhang, Bingwei Xin

**Affiliations:** 1 Wuxi Vocational Institute of Arts & Technology, Ceramic College, Yixing 214200, China; a13001716787@163.com (C.Z.); xzit12huangong@163.com (J.W.); 18851313015@163.com (F.S.); xujiacheng628@163.com (J.X.); ws19950325@163.com (S.W.); xlh961@163.com (L.X.); zhangjing_20062007@126.com (J.Z.); 2 Shazhou Professional Institute of Technology, Intelligent Manufacturing College, Zhangjiagang 215600, China; bralliar@126.com; 3 School of Environment & Energy, Zhejiang Guangsha Vocational and Technical Uninversity of Construction, Dongyang 322100, China

**Keywords:** cobalt-based catalysts, ionic liquid, deep eutectic solvent, water splitting, calcination method

## Abstract

The recent advancements of ionic liquids (ILs) and deep eutectic solvents (DESs) in the synthesis of cobalt-based catalysts for water splitting is reviewed. ILs and DESs possess unique physical and chemical properties, serving as solvents, templates, and reagents. Combined with calcination techniques, their advantages can be fully leveraged, enhancing the stability and activity of resulted catalysts. In these solvents, not only are they suitable for simple one-step calcination, but also applicable to more complex multi-step calcination, suitable for more complex reaction conditions. The designability of ILs and DESs allows them to participate in the reaction as reactants, providing metal and heteroatoms, simplifying the preparation system of cobalt phosphide, sulfide, and nitride. This work offers insights into design principles for electrocatalysts and practical guidance for the development of efficient and high-performance materials for hydrogen production and energy storage systems.

## 1. Introduction

Ionic liquids (ILs) are unique liquids composed entirely of ions without any molecular components [1,2,3]. They are typically formed by organic cations such as quaternary ammonium, quaternary phosphonium, and pyridinium ions, paired with anions like halides, tetrafluoroborate, and hexafluorophosphate, and so on. ILs remain liquid at room temperature or slightly above due to the relatively weak interactions between their cationic and anionic constituents. This characteristic allows ILs to possess unique physical and chemical properties, such as high ionic conductivity, chemical stability, and non-volatility. With the deepening of scientific research, the number of types of ILs has increased dramatically, and their structural complexity and performance diversity have improved significantly. Polymeric ILs (PILs) [4], IL crystals [5], functionalized ILs [6], and zwitterionic liquids [7] will be considered as some of the most promising reaction media. The distinctive features of ILs render them widely applicable across various domains, such as chemical synthesis [8], electrochemistry [9,10], environmental science [11], materials science [12,13], and the energy sector [14]. Especially in the field of catalysis, ILs have effectively improved the preparation achievement of catalysts, promoting the innovative development of catalysis science. As green and sustainable reaction media, ILs are of great significance in promoting the green transformation of the chemical industry and solving the problems of environmental pollution and sustainable development. Despite their promising applications, the synthesis of ILs is complex and often necessitates the use of organic solvents, which not only increases production costs but also poses potential environmental risks [15,16]. Additionally, the purification process for ILs is intricate [17].

DESs are two-component or three-component mixtures composed of hydrogen bond acceptors (HBAs) and hydrogen bond donors (HBDs) in specific stoichiometric ratios [18,19]. The freezing points of these mixtures are significantly lower than those of the individual pure components. This is because hydrogen bonds are formed between HBAs and HBDs, which lowers the overall freezing point of the system [20]. The composition of DESs can be very diverse. Common HBAs include quaternary ammonium salts and zwitterions, while typical HBDs include urea, thiourea, carboxylic acids, polyols, amino acids, sugars, trifluoroacetamide, etc. [21,22]. DESs exhibit a series of unique properties similar to those of ILs [23,24], referred to as “quasi ionic liquids”, offering diverse applications in various fields including energy storage, catalysis, and chemical synthesis.

ILs and DESs, as green solvents and functional materials, exhibit unique advantages in the field of preparing transition metal catalysts over traditional solvents. They play multiple roles in this process, serving as solvents, stabilizers, templating agents, and even reaction media [25]. ILs and DESs can finely tune the reaction environment and optimize the structures of catalysts through unique solvent effects, ion pairing, or electrostatic interactions. Moreover, by varying the types and ratios of components, the physicochemical properties of ILs and DESs can be precisely adjusted, thereby creating microenvironments suitable for different transition metal catalysts and their catalytic reactions. This optimization enhances the activity and selectivity of transition metal catalysts. Such tunability makes ILs and DESs increasingly flexible and adaptable in catalytic applications, meeting the diverse needs of various chemical reactions. The methods for synthesizing transition metal catalysts based ILs and DESs are diverse, including electrodeposition [26], solvothermal synthesis [27], microwave synthesis [28], calcination method [29], etc.

The calcination method is a thermal treatment for raw materials, heating them in an oxidative atmosphere to achieve dehydration, decarbonization, or other reactions, which is easy to operate and scale [30]. Key benefits include precise structural control and efficient purification, enhancing catalyst stability and activity. It is crucial for synthesizing efficient transition metal catalysts in energy and chemicals.

The exceptional thermal stability of ILs and DESs makes them ideal solvents for calcination techniques [29]. Compared to the traditional calcination process in conventional solvents, the IL/DES-mediated calcining synthesis strategy offers the following advantages:

Enhancing dispersion uniformity: Their superior solubility results in effectively dissolving transition metal precursors, which is a prerequisite for the uniform formation of nanomaterials.

Optimizing controllability: The physicochemical properties of such solvents (such as viscosity, density, and polarity) can be precisely controlled by adjusting their composition, thereby optimizing the calcining conditions of the catalyst and improving its performance.

Improving selectivity: In the calcination process, ILs and DESs can enhance the selectivity of reactions, reduce the occurrence of side reactions, and improve the purity of catalysts.

Achieving environmental friendliness: Under the current mainstream understanding, the low volatility and toxicity of ILs and DESs can reduce environmental pollution and comply with the development trend in green chemistry.

However, the high costs of ILs and DESs, along with their technical complexity and difficulties in recycling and reuse, may limit their widespread industrial applications and increase processing costs.

Hydrogen energy, recognized for its abundance, easy storage and transport, and high energy density, is increasingly becoming a significant force supporting global energy needs [31]. It plays a crucial role in achieving low-carbon economic transformation. Water splitting technology is the primary method of converting water into hydrogen and oxygen [32]. This process is not only clean but also, the components return to water through combustion, making it virtually harmless to the environment. The water splitting process involves two key electrochemical reactions: at the cathode, hydrogen is released through the hydrogen evolution reaction (HER), while at the anode, oxygen is generated through the oxygen evolution reaction (OER) [33,34]. However, the kinetics of both HER and OER are relatively slow, so a higher voltage (i.e., overpotential) is required to drive the reaction in practical applications, which affects the overall energy efficiency and economic viability of the system to some extent.

To overcome these challenges, researchers are actively engaged in developing novel, highly efficient electrocatalysts to reduce the required overpotential and enhance the overall efficiency of water splitting [35]. Transition metal catalysts effectively facilitate the formation of intermediates in water splitting by using their unique d-orbital electron configurations, thereby lowering activation energies and expediting reaction progress. Such catalysts not only contribute to water splitting but also offer new possibilities for achieving efficient, economical, and environmentally friendly hydrogen energy production, offering extensive applications and profound impacts in the field of clean energy.

In light of the advancements in this field, this review focuses on the synthesis of cobalt-based electrocatalysts for water electrolysis using the calcination method based on ILs and DESs. It is divided into four parts. The first part introduces the concepts, properties, applications, and advantages of ILs and DESs. Section 2 offers the preparation methods of ILs and DESs and performance evaluation of the obtained cobalt-based catalysts. The third part of this review summarizes the recent progress of ILs and DESs in the preparation of cobalt-based catalysts for water splititng utilizing mediated calcination techniques (Table 1). Regarding these solvents, not only are they suitable for simple one-step calcination, but they are also applicable to more complex multi-step calcination. This thorough analysis delves into the pivotal roles of ILs and DESs in promoting the development of efficient electrocatalysts for water splitting, offering profound insights into their mechanisms. The final part concludes the review and offers insights into the future direction of cobalt-based electrocatalysts utilizing ILs and DESs. The aim of this review is to help researchers understand and keep up with the latest advancements in cobalt-based catalysts based on ILs and DESs, inspiring innovative ideas for researchers. 

## 2. Experiments on IL/DES-Mediated Cobalt-Based Electrocatalysts for Water Splitting

### 2.1. Preparation of Common ILs and DESs

The preparation methods of ILs are diverse and can be mainly classified into two categories: direct synthesis methods and two-step synthesis methods [2].

#### 2.1.1. Conventional Synthesis Method of Ionic Liquids

(1) Direct synthesis method

The direct synthesis method involves a one-step process to generate the target IL through acid–base neutralization reactions or quaternization reactions. For example, the direct synthesis of ILs can be achieved through a nucleophilic addition reaction between tertiary amines and halogenated hydrocarbons or esters, or by utilizing the basicity of tertiary amines to neutralize acids.

(2) Two-step synthesis method

The first step of the two-step method involves preparing a halogen salt containing the target cation (such as a quaternary ammonium halide). The second step involves replacing the halogen ion with the target anion to obtain the target IL. This method is commonly used to prepare various ILs such as imidazolium, amino acid-based, and phosphonium-based ILs. For example, a quaternary ammonium halide is prepared by reacting a tertiary amine with a halogenated hydrocarbon, and then, the halogen ion is replaced with an anion such as BF_4_^−^ or PF_6_^−^ to finally obtain the target IL.

#### 2.1.2. Synthesis Method of Deep Eutectic Solvents

There are various methods for the preparation of DESs, mainly including the melting method, solution method, and direct mixing method [51].

(1) Melting method

The melting method is the most commonly used method for synthesizing DESs. This method involves mixing two or more compounds in a certain ratio and heating them above their melting points to allow interactions between the compounds.

(2) Solution method

The solution method involves dissolving the compounds in an appropriate solvent and allowing interactions between the compounds to occur by adjusting conditions, such as concentration and temperature.

(3) Direct mixing method

The direct mixing method involves directly combining two or more compounds in a specific ratio without heating or using solvents and utilizing physical methods to promote interactions between the compounds.

### 2.2. Morphology and Composition Characterization of Catalysts [52]

After successfully preparing ILs and DESs, the next step is to prepare the cobalt-based catalysts. This article mainly focuses on the calcination preparation method. Please refer to Section 3 for the preparation details. The commonly used instruments for characterizing the morphology and composition of catalysts include SEM, TEM, AFM, XRD, EDS, XPS, etc.

### 2.3. Evaluation of Catalytic Performance of Electrocatalysts for Water Splitting

Using an electrochemical workstation to perform water splitting is a common approach to evaluate the catalytic activity and stability of catalysts. A series of electrochemical testing methods are typically employed to comprehensively and accurately measure the activity, stability, and selectivity of the catalysts.

#### 2.3.1. Linear Sweep Voltammetry (LSV)

LSV testing serves as a fundamental method for evaluating the activity of electrocatalysts. In a standard three-electrode system, the prepared cobalt-based electrocatalyst is used as the working electrode. By controlling the linear variation in the potential, the curve depicting the change in current with respect to the potential (i.e., the LSV curve) is recorded. The onset potential and the overpotential required to achieve a specific current density (such as 10 mA/cm^2^, which is often considered a benchmark for the efficiency of solar water splitting) on this curve are crucial parameters for assessing the catalyst’s activity. Lower onset potentials and overpotentials indicate the higher catalytic activity of the catalyst.

#### 2.3.2. Tafel Slope Analysis

The Tafel slope reflects the logarithmic relationship between current density and overpotential, serving as a crucial indicator for evaluating the kinetic performance of catalysts. By converting the LSV curve into a Tafel plot, the Tafel slope can be calculated. A smaller Tafel slope indicates a faster kinetic process for the catalyst during water splitting, which translates to a higher charge transfer rate.

#### 2.3.3. Stability Testing

The stability of a catalyst is a significant consideration in its practical application. Commonly used stability testing methods include chronoamperometry and chronopotentiometry. In the former method, the potential of the working electrode is fixed, and the change in current over time is observed. In the latter method, the current density is fixed, and the change in potential over time is monitored. Long-duration stability tests can evaluate the durability of the catalyst during continuous catalytic processes.

#### 2.3.4. Electrochemical Impedance Spectroscopy (EIS)

EIS testing is employed to analyze electrochemical parameters such as charge transfer resistance and solution resistance at the interface between the catalyst and the electrolyte. By fitting the EIS spectra, we can obtain the value of charge transfer resistance. A smaller value indicates that charge transfer at the interface between the catalyst and the electrolyte is more facile, resulting in the higher catalytic efficiency of the catalyst.

#### 2.3.5. Electrochemical Specific Surface Area (ECSA)

For the same catalytic material, a larger electrochemical specific surface area indicates that more active sites can be exposed, and the catalytic activity of the catalyst is better. First, the double-layer capacitance (*C*_dl_) of the catalyst is measured. When the scanning rate remains unchanged, the change in potential with time is a constant within a certain potential range. When plotting the difference in double-layer current at different scanning rates against the scanning rate, a straight line can be obtained, and half of its slope is the double-layer capacitance *C*_dl_. ECSA is the ratio of the double-layer capacitance of the tested electrode to the double-layer capacitance of the ideal smooth oxide electrode.

## 3. IL/DES-Mediated Synthesis of Co-Based Catalysts Using Calcination Method

### 3.1. One-Step Calcination Method

The calcination method can be roughly divided into single-step calcination and multi-step calcination. Single-step calcination refers to completing all necessary chemical reactions and physical changes in a single step to prepare the desired material [53]. This process simplifies operations, enhances product consistency, reduces quality variations, saves energy, and reduces emissions. It is consistent with the concept of green chemistry [54].

The unique properties of ILs and DESs can be combined with the advantages of calcination [54]. So, IL/DES-mediated single-step calcination is a favored synthesis strategy, as it simplifies synthesis steps, promotes precursor conversion, regulates product structure, and has environmental friendliness and sustainability. By directly dissolving cobalt salts in ILs or DESs and subsequently calcining the resulting mixture, cobalt-based catalysts can be obtained. Li et al. calcinated a ChCl/urea/gluconic acid DES containing Co(NO_3_)_2_·6H_2_O, and then successfully prepared cobalt nanoparticles supported on nitrogen-doped porous carbon (NPC), naming it Co@NPC [36]. In this study, the DES played multiple critical roles. It provided three-dimensional structural support, aiding in the formation of ordered pore structures in the calcination process, thereby enhancing the specific surface area and porosity of the catalyst. The thermal stability of DESs at high temperatures helps maintain the structural integrity of the catalyst. Additionally, gluconic acid in DESs can be converted into NPC during the calcination process, which not only exhibits good electrical conductivity to enhance electrochemical performance but also serves as a carrier for the catalyst, avoiding the additional carbon source. The interactions between the components of a DES and the cobalt source facilitate the formation of uniform cobalt nanoparticles and the dispersion and stability of these cobalt nanoparticles. The prepared Co@NPC exhibited excellent HER performance, whether in acidic (*η*_10_ = 215) or alkaline (*η*_10_ = 274) environments. In addition, Co@NPC also showed outstanding water splitting performance (requiring a potential of 1.74 V at a current density of 10 mA cm^−2^) and glucose decomposition performance (reaching a potential of 1.56 V at a current density of 10 mA cm^−2^).

**Figure 1:**
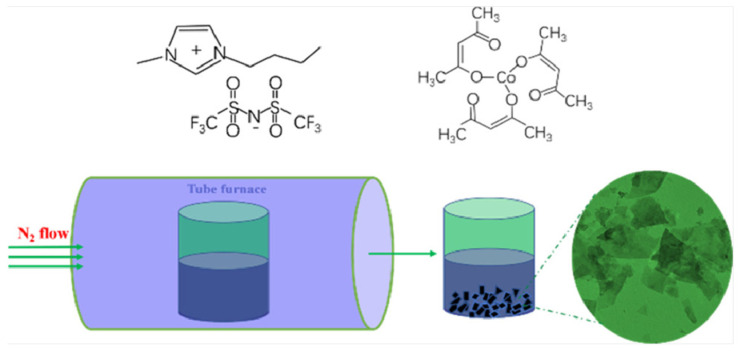
Molecular structures of [BMIM][NTf_2_] and Co(acac)_3_ and schematic illustration of the formation of the CoO electrocatalysts. Reprinted with permission from ref. [37], copyright 2018 ACS.

In order to further enhance the catalytic performance of catalysts, the screening of media has always been a research focus. The use of ILs as media for the preparation of cobalt-based catalysts has garnered consistent attention. Our research team successfully prepared ordered CoO nanosheets by directly calcining the mixture of [BMIM]Tf_2_N (1-butyl-3-methylimidazolium triflimide) and Co(acac)_3_ (Figure 1) [37]. It not only has intermolecular interactions and spatial effects, but also, the [BMIM]^+^ ion has the ability to self-assemble into polymeric supramolecular structures. CoO particles began to form and gradually aggregate together during the initial stages of the growth process, and then exhibited sheet-like structures, finally forming the ordered CoO nanosheets. The prepared CoO nanosheets offered excellent OER performance in 1M KOH solution with an *η*_10_ of 320 mV and a Tafel slope of 70 mV dec^−1^. Similarly, in [1-vinylimidazolium]HSO_4_, the addition of CoCl_2_ followed by burning under an inert atmosphere yielded a series of porous carbon materials doped with Co, nitrogen, and sulfur, referred to as Co_3_O_4_-N/S [38]. Co_3_O_4_-N/S demonstrated excellent stability up to 8 h without any decay in catalytic current for OER.

To further regulate the catalytic performance of cobalt-based catalysts, cobalt-based bimetallic catalysts attract widespread attention. Adding a second metal salt to the IL/DES-Co system and subsequently applying calcination techniques is an effective strategy for obtaining cobalt-based bimetallic catalysts. CoV_2_O_6_ with an octahedral structure was successfully synthesized through introducing cobalt and vanadium salts into a ChCl/malonic acid DES and reacting it for 2 h at a relatively low temperature of 500 °C [39]. Experiments show that in a non-DES system, CoV_2_O_6_ can be obtained by high-temperature pyrolysis at 720 °C for 40 h. This work demonstrated that a DES could not only modulate the morphology of CoV_2_O_6_ to achieve the desired regular octahedral shape but also significantly reduced the activation energy required for the reaction, thereby greatly lowering the reaction temperature and shortening the reaction time. The DES-derived octahedral CoV_2_O_6_ was an excellent electrocatalyst for OER, exhibiting a low overpotential of 324 mV at 10 mA cm^−2^.

With the development of green chemistry, simplifying the reaction system has become a hot topic of research. Therefore, strategies that directly utilize ILs or DESs as metal sources are drawing increasing interest [55]. The metal components can be designed as anions of ILs, participating directly in the reactions. Currently, many reports have documented Co-based ILs with anions such as CoCl_2_Br_2_^2−^, including N,N-bis(4-(methoxycarbonyl)benzyl)-N-methyl-D-glucamine dibromodichlorocobaltate(II) ([MBMG]_2_CoCl_2_Br_2_) [40], CoCl_2_-EMIC (1-ethyl-3-methylimidazolium chloride) [56], [ChCl]_2_CoCl_4_ [57], [P_6614_]_2_CoCl_4_ [41], etc. This means that the reaction system is pure, decreasing impurities and helping to enhance the purity of the products. To further improve the conductivity of target products, carbon nanotubes (CNTs) are often added. By calcining a mixture of CNTs and [MBMG]_2_CoCl_2_Br_2_ IL at a low temperature of 300 °C, CoP/CNTs were successfully synthesized through a phosphidation process utilizing NaH_2_PO_2_ [40]. It was observed that CoCl_2_Br_2_^2−^ reacted with NaH_2_PO_2_ to form CoP, while [MBMG]_2_⁺ transformed into amorphous carbon. The in situ introduction of amorphous carbon enhanced the electrical conductivity of CoP and improved its electron transfer capabilities. Experimental results confirmed that CoP/CNTs was a highly active HER electrocatalyst, with an onset potential of just 55 mV, an overpotential of 135 mV at 10 mA cm^−2^, and a Tafel slope of 58 mV dec^−1^.

**Figure 2:**
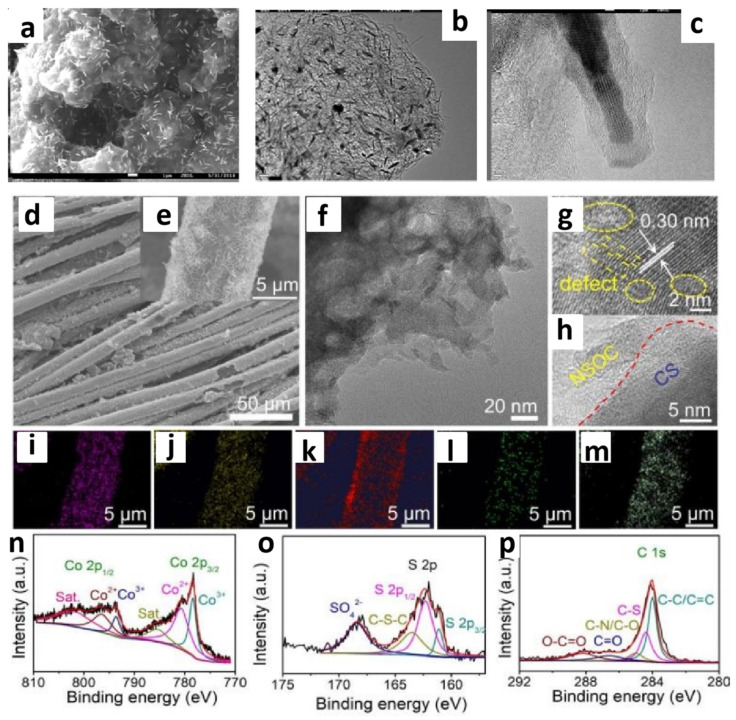
FE-SEM image of Co_2_P/CNTs (**a**), TEM image of Co_2_P/CNTs (**b**,**c**). Reprinted with permission from ref. [41], copyright 2021 Elsevier. Typical FESEM images (**d**,**e**), TEM image (**f**), HRTEM images (**g**,**h**), (Co, S, C, N, O) EDX mapping (**i**–**m**), Co 2p, S 2p, and C 1s high-resolution XPS spectra of the N,S,O-C/Co_9_S_8_ (**n**–**p**). Reprinted with permission from ref. [42], copyright 2021 RSC.

Furthermore, a more advanced design is to allow ILs or DESs to serve as both a cobalt source and a phosphorus source. Li’s research group successfully employed trihexyl(tetradecyl)phosphonium CoCl_4_ ([P_6614_]_2_CoCl_4_) as a dual source of phosphorus and cobalt. By mixing this compound with CNTs and subsequent calcination, they prepared Co_2_P/CNT composites (Figure 2a–d) [41]. The experimental results demonstrated that [P_66614_]_2_[CoCl_4_] was capable of forming Co_2_P without the addition of any other reagents. The involved CNTs increased electrical conductivity and aided in the formation of cobalt phosphide. In traditional methods, multiple steps may be required to separately synthesize the phosphorus and cobalt sources before combining them with CNTs. However, with [P_6614_]_2_CoCl_4_, it can be directly mixed with CNTs, allowing for the target catalyst to be obtained in a single step or with just a few reactions, greatly simplifying the catalyst preparation process. In acidic solutions, Co_2_P/CNT exhibited outstanding HER activity.

The development of Co-based ILs has significantly promoted the research into Co-based DESs. CoCl_2_·6H_2_O, serving as an efficient HBA, is mixed with HBDs such as alcohols, carboxylic acids, and amides in specific molar ratios to form DESs [58]. Under suitable conditions, they can simultaneously release metal ions and other heteroatoms, thereby facilitating the construction of cobalt-based catalysts. The mixture of a CoCl_2_·6H_2_O/thiourea DES was coated onto carbon cloth [42]. After calcination, a sulfur, nitrogen, and oxygen tri-doped carbon/Co_9_S_8_ composite (N,S,O-C/Co_9_S_8_) was obtained. In this process, both HBD and HBA in the DES participated in the reaction, providing sources of Co, S, and N, respectively. By adjusting the ratio of HBD and HBA, the composition and properties of N,S,O-C/Co_9_S_8_ could be tailored. The molecular structure of the DES may have influenced the microstructure and morphology of the final composite materials, which resulted in the catalyst exhibiting excellent electrocatalytic activity with low overpotential and high stability for HER with different pH values (Figure 2e–p).

Transition metal nitrides are comparatively more difficult to prepare than sulfides and phosphides. Common nitrogen sources used for the preparation of metal nitrides include NH_3_, cyanides (such as KCN or NH_4_CNO), and organic amines [59]. However, this method has some drawbacks, including slow reaction rates, low product purity, and the toxicity and dangerousness of NH_3_. Recently, ILs and DESs have been designed to contain specific functional groups involving N. They can supply N and operate lower temperatures, thereby avoiding the high-temperature conditions typically required when using N_2_ directly. Since ILs and DESs can provide N at the molecular level, they enable precise control over the nitridation reaction, fostering the formation of specific crystalline structures and morphologies. The high thermal stability and tunable polarity of ILs and DESs contribute to enhanced reaction efficiency and product quality. These advantages enhance the electrochemical or catalytic performance of products.

**Figure 3:**
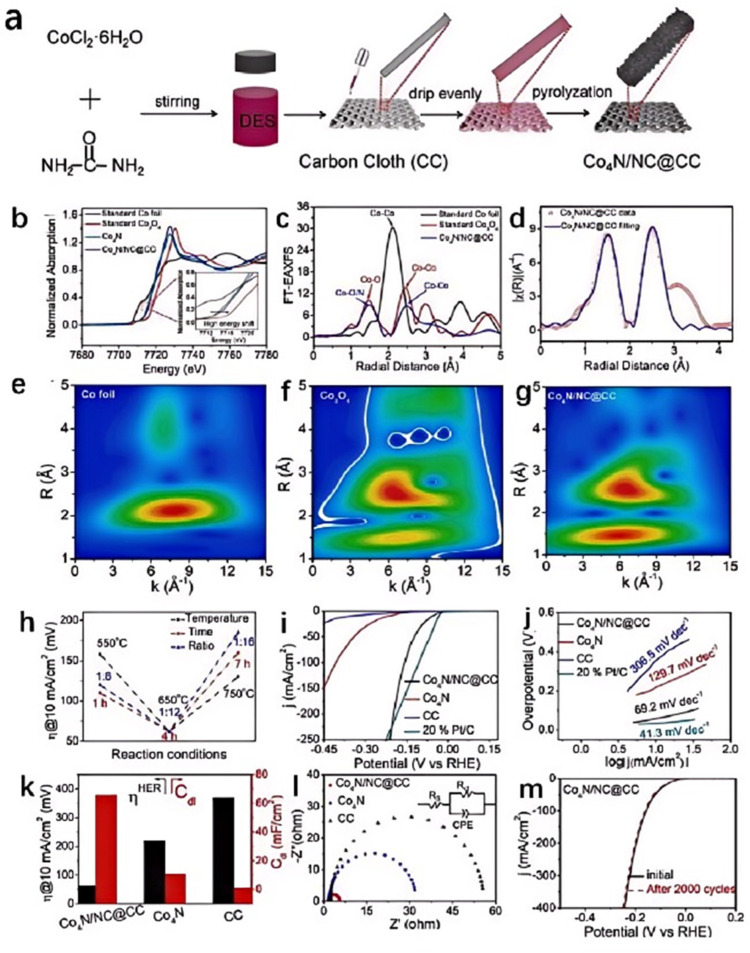
Schematic illustration of the synthesis procedure of Co_4_N/NC self-supporting electrode (**a**), the normalized XANES spectra for Co K-edge of Co_4_N/NC and the standard samples (**b**), FT-EXAFS spectra in the R space of Co_4_N/NC and the standard samples (**c**), corresponding EXAFS fitting curves of Co_4_N/NC in the R space (**d**), WT-EXAFS of (**e**) Co foil, Co_3_O_4_ (**f**), and Co_4_N/NC (**g**), a comparison of the overpotential of the Co_4_N/NC@CC electrode obtained in different conditions (**h**), LSV curves (**i**), Tafel plots (**j**), the comparison between the overpotential and *C*_dl_ (**k**), and Nyquist plots of different catalysts in 1.0 M KOH (**l**), LSV curves for stability tests (**m**). Reprinted with permission from ref. [44], copyright 2023 Elsevier.

Urea, as an organic nitrogen source, when mixed with CoCl_2_ in a certain proportion, can prepare CoCl_2_/urea DES. Different reaction conditions can lead to different products. Our research group obtained two-dimensional (2D) [Co-(NH_3_)_4_CO_3_]Cl nanosheets all-in-one by pyrolyzing this DES at 180 °C for 21 h [43]. The reaction conditions led to complex chemical reactions within the DES, such as the decomposition of urea at high temperatures to produce NH_3_ and CO_2_. The latter reacted with certain intermediates to form CO_3_^2−^, while Co^2+^ coordinated with NH_3_ to form complexes. The activated [Co-(NH_3_)_4_CO_3_]Cl/Ni foam electrocatalyst was prepared by applying a chronoamperometry test in 1.0 M KOH. After the activation process, the 2D [Co-(NH_3_)_4_CO_3_]Cl nanosheets converted to amorphous CoOOH, resulting in good catalytic performance with 291 mV to achieve 10 mA cm^−2^ and a satisfactory Tafel slope (65 mV dec^−1^). Moreover, the long-term stability results indicate its good durability for OER. Zhang dripped a CoCl_2_·6H_2_O/urea DES on a carbon cloth and successfully prepared a composite of nitrogen-doped carbon (NC) with ultrathin Co_4_N nanosheets (Co_4_N/NC) on the carbon cloth through calcination (Figure 3a–g) [44]. During the pyrolysis process, CoCl_2_ and urea in the DES interacted with each other, forming NC and Co_4_N compounds. The DES provided a homogeneous reaction environment that allowed CoCl_2_·6H_2_O and urea to react within the same medium. This uniform reaction environment helped control the morphology and structure of the products, such as the formation of ultrathin Co_4_N nanosheets. The specific components in the DES might interact with CoCl_2_·6H_2_O to facilitate the formation and dispersion of Co_4_N. Additionally, these components can also interact with carbon cloth or Co_4_N to form strong interfacial bonding, which favored the rapid transfer of electrons between Co_4_N and NC. Nitrogen doping could alter the electronic structure of carbon, enhancing the conductivity of carbon materials and thereby improving their electrochemical performance. In Co_4_N/NC, the strong interaction between Co_4_N and NC significantly accelerated the transport of electrons at the interface, while the ultrathin nanostructure increased the number of catalytically active sites, thereby significantly enhancing the catalytic performance of Co_4_N/NC, which required only 62, 98, and 60 mV overpotentials to achieve a current density of 10 mA cm^−2^ in alkaline, neutral, and acidic electrolytes for the HER, respectively (Figure 3h–m).

The M-N-C catalyst has been widely recognized as one of the most promising catalysts for OER due to its excellent activity and durability [60]. ILs, such as 1-ethyl-3-methyl imidazolium dicyanamide (EMIM-dca) [61] and [Bmim][BF_4_] [62], were successively reported to be calcined with cobalt salt in the preparation of Co-N-C catalysts. Furthermore, PILs are found to be even superior precursors for M-N-C catalysts due to the inherent advantages of both polymers and ILs [45,63]. PILs contain high levels of N and C elements. Their polymeric structure enables a more uniform and stable distribution of N and C elements, which contributes to the formation of more numerous and stable active sites within the catalyst. For example, as a renewable resource, cellulose imparts cellulose-based PILs with excellent green and biodegradable properties [63]. In addition, by altering the degree of polymerization, ion species, and functional groups of PILs, their physicochemical properties such as viscosity, solubility, and thermal stability can be tuned to meet the requirements of different catalytic reactions. During the pyrolysis process of the PIL-containing nitrate group, NO_3_^−^ is capable of vigorously releasing gasses, thereby facilitating the formation of porous structures [45]. Upon mixing with Co(NO_3_)_2_ and undergoing a one-pot thermal treatment process, porous Co-N Co-doped carbon nanosheets (porous Co-N-C) were successfully synthesized. They exhibited remarkable performance in the OER, requiring only an overpotential of 400 mV to achieve a current density of 10 mA·cm^−2^ with a small Tafel slope of 127.4 mV/dec.

When ILs are used as media in the preparation of catalysts, they are often required in relatively large quantities. This not only leads to waste but also complicates the subsequent processing and causes environmental pollution. Based on this, novel Co-containing DESs were designed and synthesized, such as Co(NO_3_)_2_·6H_2_O/[Bmim]Br [64], CoCl_2_·6H_2_O/[Bmim]BF_4_ [65], and so on. Experiments have proven that Co(NO_3_)_2_·6H_2_O/[Bmim]Br is not a mixture of IL and Co(II) salt. Strong bonding is formed between Co(NO_3_)_2_·6H_2_O and [Bmim]Br, which can provide stable microenvironments for metals and promote a uniform distribution of Co and N atoms in electrocatalysts.

As DESs are inherently designable, various types of DESs can be prepared by employing a multitude of HBAs and HBDs. For example, FeCl_3_·6H_2_O, CoCl_2_·6H_2_O, and NiCl_2_·6H_2_O can form multiple HBAs. Using this type of HBA in combination with an HBD, such as L-cysteine, can yield a novel DES. Through a simple one-step calcination process, N,S-doped NiCoFe alloy could be directly synthesized from this DES [46]. In this process, the DES played multiple roles simultaneously: as a solvent, template, ligand, metal source, nitrogen source, and sulfur source. The templating effect of the DES not only endowed the catalyst with a porous structure and high specific surface area but also ensured the uniform distribution and synergistic action of Ni, Co, and Fe. These structural features, including multi-metal active sites, high specific surface area, and porosity, enabled the resulting N,S-doped NiCoFe to exhibit excellent OER performance under alkaline conditions, with low overpotential and a small Tafel slope. This work demonstrates a new strategy for developing high-performance multifunctional electrocatalysts by designing specific DESs, offering valuable insights into energy conversion and storage research.

### 3.2. Multi-Step Calcination Method

The one-step calcination technique is simple and quick. However, for more complex preparations, it is necessary to first synthesize the precursors before calcining them. This technique is generally referred to as multi-step calcination [66].

Step One—Preparation of precursor materials: In the first step, appropriate raw materials are usually selected and mixed in a certain proportion. These raw materials may include metal salts, organics, oxides, etc. Through mixing and calcination, the required precursor materials can be prepared.

Step Two—Calcination steps: In the second step, the precursor materials obtained from the first step are subjected to calcination. During the calcination process, factors such as temperature, time, and atmosphere have significant impacts on the properties of the final product.

In comparison with single-step calcination, multi-step calcination offers better control over the structure and properties of the final product. By carefully adjusting key parameters such as temperature, atmosphere, and holding time at different calcination stages, this technique can precisely shape the growth and aggregation characteristics of raw material particles, thereby preparing materials with specific sizes, shapes, and pore structures, ultimately obtaining products that meet the requirements. This precise control over morphology is particularly critical in various high-tech fields, including catalysts, battery materials, ceramic manufacturing, and nanotechnology, as it directly relates to the optimization of material performance and the maximization of application characteristics.

**Figure 4:**
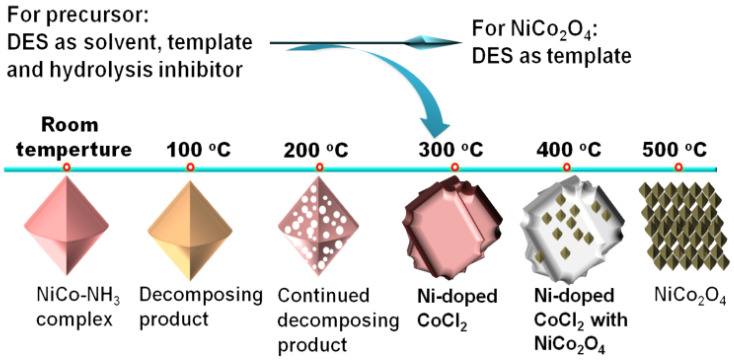
Schematic diagram of the synthesis process of NiCo-NH_3_ complex precursor and NiCo_2_O_4_. Reprinted with permission from ref. [47], copyright 2022 Elsevier.

Our research group developed a two-step method for preparing octahedral NiCo_2_O_4_ [47]. In the first step, we mixed aqueous ammonia, CoCl_2_, and NiCl_2_ into a DES composed of ChCl and glycerol. With assistance of the DES, a NiCo-NH_3_ complex was easily formed at room temperature. This indicates that the DES is capable of effectively suppressing its hydrolysis of the NiCo-NH_3_ complex. It is well known that the NiCo-NH_3_ complex is prone to hydrolysis. So, the synthesis of this complex in an aqueous solution would typically require a specific low-temperature environment and the presence of hydrolysis inhibitors. In the second step, we calcined the NiCo-NH_3_ complex, which could be fully oxidized in just 15 min, ultimately yielding pure NiCo_2_O_4_ (Figure 4). This method was not only straightforward to operate but also significantly improved the efficiency of preparation. The two-step method for preparing octahedral NiCo_2_O_4_ not only simplifies the operation compared to traditional preparation methods but also significantly improves the efficiency of synthesis. In our study on the impact of calcination temperature and duration on the morphology and composition, we found that the DES used not only facilitated the formation of precursors and oxides but also could regulate the morphology of intermediates and products. Due to the synergistic action of Ni and Co, as well as the unique octahedral structure, the prepared NiCo_2_O_4_ exhibited outstanding OER performance and excellent stability. In a 1.0 M KOH electrolyte, the *η*_10_ and Tafel slope of NiCo_2_O_4_ were only 320 mV and 67 mV dec^−1^, respectively_._ This research deepens our understanding of the role of DES in the synthesis of inorganic materials, particularly in its application for controlling material structure and properties.

**Figure 5:**
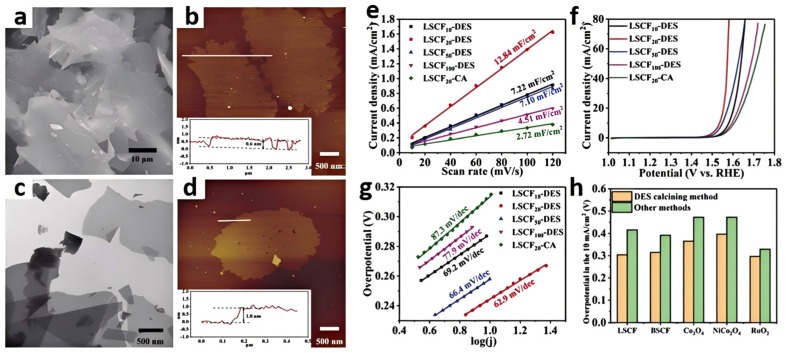
SEM (**a**) and AFM images (**b**) of 2D carbon flake templates from the calcined glucose/urea DES; TEM (**c**) and AFM images (**d**) of 2D carbon flake templates with metal species. The insets of AFM images show the corresponding height measurements, Δj of the La_0.5_Sr_0.5_Co_0.8_Fe_0.2_O_3_ plotted against scan rates (**e**), LSVs for different La_0.5_Sr_0.5_Co_0.8_Fe_0.2_O_3_ in the 1.0 M KOH electrolyte (**f**). Scan rate: 5 mV s^−1^. (**g**) Tafel plots for different La_0.5_Sr_0.5_Co_0.8_Fe_0.2_O_3_. Comparison of the overpotentials at 10 mA cm^−2^ for La_0.5_Sr_0.5_Co_0.8_Fe_0.2_O_3_, Ba_0.5_Sr_0.5_Co_0.8_Fe_0.2_O_3_, Co_3_O_4_, NiCo_2_O_4_, and RuO_2_ synthesized by the DES calcining method and other classical methods (the citric acid combustion method for La_0.5_Sr_0.5_Co_0.8_Fe_0.2_O_3_ and Ba_0.5_Sr_0.5_Co_0.8_Fe_0.2_O_3_ and the wet chemical method for Co_3_O_4_, NiCo_2_O_4_, and RuO_2_) (**h**). Reprinted with permission from ref. [48], copyright 2023 Elsevier.

Other interesting work has described the preparation of two-dimensional sheet-like La_0.5_Sr_0.5_Co_0.8_Fe_0.2_O_3_ through the DES calcining method (Figure 5a–d) [48]. The synthesis of oxides undergoes several stages. Firstly, suitable amounts of metal salt precursors were dissolved in a glucose/urea DES. Then, the metal-containing DES solution was heated in a household microwave oven to form the liquid which turned into black foam. Afterward, the foam was calcined under a N_2_ atmosphere. During this process, the DES acted as a carbon source, and the 2D graphene flakes loaded with metal species were produced. Finally, the precursor was calcined at high temperatures in air, where the metal species underwent oxidation, transforming into the desired two-dimensional sheet-like oxide composition. This method could also be applied to obtain a series of two-dimensional metal oxides, such as Ba_0.5_Sr_0.5_Co_0.8_Fe_0.2_O_3_, Co_3_O_4_, NiCo_2_O_4_, and RuO_2_. The use of a DES enhanced the thermal stability and conductivity of the final catalyst, as the introduction of a carbon framework improved the electron transport pathways. The obtained La_0.5_Sr_0.5_Co_0.8_Fe_0.2_O_3_ exhibited good OER performance (*η*_10_ = 304 mV). The presence of a DES helps control the morphology of the final products. By adjusting the calcination conditions, the size and distribution of the metal oxides can be precisely controlled, which has a direct impact on the electrochemical performance of the catalysts (Figure 5e–h).

**Figure 6:**
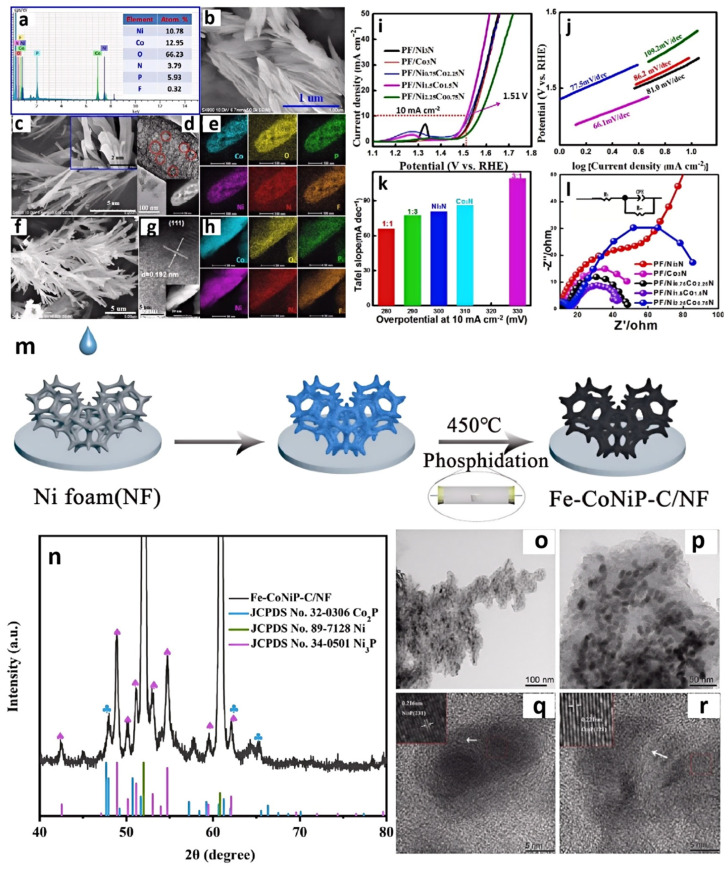
EDS spectrum and atomic concentrations (at%) of the N, P, and F species in N,P-Ni_1.5_Co_1.5_N (**a**), SEM image of N,P-Ni_1.5_Co_1.5_N catalyst (**b**), SEM (**c**), HR-TEM (**d**), HAADF-STEM (inset in **d**), and mapping results of heteroatom-doped Ni-Co (1:1) precursor (**e**). SEM (**f**), HR-TEM (**g**), HAADF-STEM (inset in **e**), and mapping results of N,P-Ni_1.5_Co_1.5_N (**h**), LSV curves (**i**), and Tafel slopes (**j**) of N,P-/Co_x_Ni_3−x_N catalysts for OER. Comparison of *ƞ*_10_ values (potentials required to reach 10 mA cm^−2^) for various catalysts (Ni/Co = 1:1, 1:3, 1:0, 0:1, and 3:1) (**k**). EIS Nyquist plots at 0.6 V for PF/Co_x_Ni_3−x_N catalysts (**l**). Reprinted with permission from ref. [49], copyright 2017 Wiley. Preparation of Fe-CoNiP-C/NF (**m**). XRD patterns of Fe-CoNiP-C/NF (**n**), TEM images of Fe-CoNiP-C/NF (**o**–**r**). Reprinted with permission from ref. [50], copyright 2023 Wiley.

Zhang et al. developed a facile strategy for the synthesis of well-defined phosphorus, fluorine co-doped Ni_1.5_Co_1.5_N hybrid nanorods by using [BMIM]PF_6_ (Figure 6a) [49]. They mixed [BMIM]PF_6_ into a Co^2+^:Ni^2+^ solution to form a precursor, where [BMIM]PF_6_ helped introduce nitrogen and phosphorus (Figure 6b–h). This initial nitridation may have been insufficient to achieve the desired nitrogen and phosphorus content, or it may have resulted in uneven nitridation. So, secondary nitridation in ammonia was undergone and yielded N,P-Ni_1.5_Co_1.5_N. The secondary nitridation could ensure a more complete nitridation reaction and better control over the doping levels of N and P. More uniform nitrogen and phosphorus doping can alter the electronic structure of materials, enhancing their conductivity and catalytic activity. IL could also regulate the morphology of the product. The bimetallic nitride Ni_1.5_Co_1.5_N synthesized without IL exhibited lamellar morphology. However, after adding IL into the system, the morphology of the catalyst changed from nanosheets to nanorods. After being calcined at high temperature in NH_3_, numerous layered structures of Co_2_N_0.67_ attached onto the surface of the Ni_3_N nanorods and formed a heterojunction structure of Ni_3_N/Co_3_N hybrids. N,P-Ni_1.5_Co_1.5_N could obtain high OER activity, requiring only a 280 mV overpotential for a current density of 10 mA cm^−2^ and a Tafel slope of 66.1 mV dec^−1^ (Figure 6i–l).

Phosphides can be prepared using similar methods. Li and colleagues firstly modified nickel foam with trihexyl(tetradecyl)phosphonium tetrachloroferrate ([P(C_6_H_13_)_3_C_14_H_29_][FeCl_4_]) and trihexyl(tetradecyl)phosphonium tetrachlorocobaltate ([P(C_6_H_13_)_3_C_14_H_29_]_2_[CoCl_4_]) ILs [50]. This helped improve the surface characteristics of the nickel foam, making it more suitable for subsequent phosphidation treatment. In the second step, the mixture was heated with Na_2_HPO_4_ at a temperature of 450 °C to perform phosphorization, obtaining Fe-CoNiP (Figure 6m–r). The Fe-CoNiP-C/NF demonstrated excellent OER activity, requiring only 355 and 424 mV of overpotential to achieve current densities of 50 and 100 mAcm^−2^, respectively. Moreover, it exhibited good stability for over 30 h at a current density of 100 mA cm^−2^.

## 4. Summary and Outlook

This review summarizes the recent progress made in utilizing ionic liquids (ILs) and deep eutectic solvents (DESs) for the synthesis of cobalt-based catalysts aimed at water splitting. ILs and DESs, owing to their unique physical and chemical properties, have emerged as versatile solvents, templates, and reagents in the catalyst preparation process. By integrating these solvents with calcination techniques, researchers have achieved precise control over the catalyst’s crystal structure, particle size distribution, and porosity, leading to the enhanced stability and activity of the resulting cobalt-based catalysts. Notably, these solvents are adaptable to both simple one-step and more complex multi-step calcination procedures, allowing for the optimization of catalyst properties under diverse reaction conditions. Furthermore, the design flexibility of ILs and DESs enables them to act as reactants, introducing metal and/or heteroatoms as well as simplifying the synthesis routes for cobalt phosphide, sulfide, and nitride catalysts.

The use of ILs and DESs has opened up a promising avenue for catalyst preparation. However, when choosing ILs and DESs to prepare catalysts, their high cost and potential toxicity need to be considered. Although they are generally regarded as environmentally friendly substitutes, not all types of ILs and DESs are completely non-toxic or biodegradable. Some may contain environmentally harmful components. There are no definitive data on the potential impact of their large-scale use and disposal on the environment. Meanwhile, some ILs and DESs may exhibit lower stability under specific reaction conditions. For example, they may decompose or lose their original functions in high-temperature or strong acid/alkali environments [67], thereby affecting the performance of the catalyst.

To further improve the performance of cobalt-based catalysts, future research may focus on several aspects: firstly, combining advanced characterization techniques and theoretical calculations to deeply understand the calcination mechanism mediated by ILs/DESs. Through functional design, the stability of ILs and DESs under specific reaction conditions can be improved, such as introducing specific functional groups to enhance their high temperature resistance, acid and alkali resistance, and other properties, thereby guiding the rational design and efficient preparation of catalysts. Secondly, researchers should design and synthesize more excellent ILs and DESs. Based on the principle of sustainability, ILs and DESs can be directly used as reaction reagents to simplify the reaction operation steps and reduce emissions.

## Figures and Tables

**Table 1:** Table of water splitting performance of IL/DES-derived cobalt-based catalysts prepared by calcination technology.

Catalyst	Applied IL/DES	Catalytic Performance						Ref.
		HER			OER			
		Electrolyte	*ƞ*(mV)@Current Density (mA cm^−2^)	Tafel Slope (mV dec^−1^)	Electrolyte	*ƞ*(mV)@Current Density (mA cm^−2^)	Tafel Slope (mV dec^−1^)	
Co@NPC	ChCl/urea/gluconic acid	0.5 M H_2_SO_4_	215@10	70	--	--	--	[36]
Co@NPC	ChCl/urea/gluconic acid	1 M KOH	274@10	91	1 M KOH	430@10	87	[36]
CoO	[BMIM]Tf_2_N	--	--	--	1 M KOH	320@10	70	[37]
Co_3_O_4_-N/S	[1-vinylimidazolium]HSO_4_	--	--	--	1 M KOH	350@10	67	[38]
CoV_2_O_6_	ChCl/malonic acid	--	--	--	1 M KOH	324@10	--	[39]
CoP/CNT	[MBMG]_2_CoCl_2_Br_2_	0.5 M H_2_SO_4_	135@10	58	--	--	--	[40]
Co_2_P/CNT	[P_66614_]_2_CoCl_4_	0.5 M H_2_SO_4_	150@10	47	--	--	--	[41]
N,S,O-C/Co_9_S_8_	CoCl_2_·6H_2_O/TU	1 M KOH	53@10	45.3	--	--	--	[42]
N,S,O-C/Co_9_S_8_	CoCl_2_·6H_2_O/TU	1 M PBS	103@10	113.7	--	--	--	[42]
N,S,O-C/Co_9_S_8_	CoCl_2_·6H_2_O/TU	0.5 M H_2_SO_4_	68@10	31	--	--	--	[42]
CoOOH	CoCl_2_/urea	--	--	--	1 M KOH	291@10	65	[43]
Co_4_N/NC@CC	CoCl_2_·6H_2_O/urea	0.5 M H_2_SO_4_	62@10	--	--	--	--	[44]
Co_4_N/NC@CC	CoCl_2_·6H_2_O/urea	1 M PBS	98@10	--	--	--	--	[44]
Co_4_N/NC@CC	CoCl_2_·6H_2_O/urea	1 M KOH	60@10	69.2	--	--	--	[44]
Co-N-C	hydrolyzed vinyl imidazolium nitrate				1 M KOH	400@10	127.4	[45]
N,S-NiCoFe	FeCl_3_·6H_2_O/CoCl_2_·6H_2_O/NiCl_2_·6H_2_O/L-cysteine	--	--	--	1 M KOH	251@10	58	[46]
NiCo_2_O_4_	ChCl/glycerol	--	--	--	1 M KOH	320@10	61	[47]
La_0.5_Sr_0.5_Co_0.8_Fe_0.2_O_3_	Glucose/urea DES	--	--	--	1 M KOH	304@10	62.9	[48]
Ba_0.5_Sr_0.5_Co_0.8_Fe_0.2_O_3_	Glucose/urea DES	--	--	--	1 M KOH	315@10	--	[48]
NiCo_2_O_4_	Glucose/urea DES	--	--	--	1 M KOH	365@10	--	[48]
Co_3_O_4_	Glucose/urea DES	--	--	--	1 M KOH	397@10	--	[48]
P,F-Ni_1.5_Co_1.5_N	[BMIM]PF_6_	--	--	--	1 M KOH	280@10	66.1	[49]
Fe-CoNiP	[P(C_6_H_13_)_3_C_14_H_29_] [FeCl_4_], [P(C_6_H_13_)_3_C_14_H_29_]_2_ [CoCl_4_]	--	--	--	1 M KOH	355@50 424@100	45	[50]

## Data Availability

Not applicable.
